# Supplementary material for: Height development and multiple bone health indicators in children aged 2–12 years with Duchenne muscular dystrophy (DMD)
Source: PLoS One. 2025 Jan 10;20(1):e0316938. doi: 10.1371/journal.pone.0316938 (PMC11723629; doi:10.1371/journal.pone.0316938)
Supplement: Attachment — Submitted filename: pone DMD review.docx [file pone.0316938.s001.docx]

**Title:** Review of Manuscript “**Height development and multiple bone health indicators in children aged 2-12 years with Duchenne muscular dystrophy (DMD)”**

Bone health impairment and short stature are significant issues faced by children with Duchenne Muscular Dystrophy (DMD), regardless of whether they are undergoing glucocorticoid treatment. This manuscript is valuable for its aim to shed light on these problems. However, there are several critical points that require clarification:

1. The authors mention that height and weight were measured using standard equipment. It would be beneficial to clarify what is meant by "standard equipment." Additionally, in ambulatory patients, accurate height measurement can be challenging in DMD children due to factors such as scoliosis, increased lumbar lordosis, and contractures. Therefore, it is important to specify the percentage of patients with scoliosis, contractures, and increased lumbar lordosis. For non-ambulatory patients, it is evident that height measurement cannot be performed using standard equipment, so a detailed explanation is necessary.
2. The abbreviation "MDT" in line 114 should be explained.
3. Bone Mineral Density (BMD) is typically expressed in units of g/cm². The authors should clarify their use of the mg/cc unit.
4. The authors report a vertebral fracture (VF) rate of 0% in their patients. However, asymptomatic vertebral fractures can occur in 30-50% of DMD children, especially those on glucocorticoid therapy. Therefore, annual (or more frequent if symptoms are present) monitoring with lateral spinal radiographs is recommended. The manuscript does not clearly state the frequency of routine follow-ups, nor whether asymptomatic vertebral fractures were actively investigated through lateral spinal radiographs. Without this monitoring, claiming a 0% VF rate may not be accurate.
5. Are there any patients with long bone fractures, diagnosed osteoporosis, or those receiving bisphosphonate treatment?
6. In Figure 1-d, the authors should explain why height growth velocity is so low at age 3 in the DMD population.
