# Supplementary material for: Height development and multiple bone health indicators in children aged 2–12 years with Duchenne muscular dystrophy (DMD)
Source: PLoS One. 2025 Jan 10;20(1):e0316938. doi: 10.1371/journal.pone.0316938 (PMC11723629; doi:10.1371/journal.pone.0316938)
Supplement: Attachment — Submitted filename: Response to Reviewers.docx [file pone.0316938.s002.docx]

**Response Letter**

Dear editors and reviewers,

Thank you for the detailed review and the precious opportunity to revise our manuscript titled “Height development and multiple bone health indicators in children aged 2-12 years with Duchenne muscular dystrophy (DMD)” (ID: PONE-D-24-27979). We are very grateful for your constructive comments and suggestions, which are very valuable and helpful for improving our manuscript. In the following, the responses to all the comments are provided one by one.

We have tried our best to make all the revisions clear, and we hope that the revised manuscript can satisfy the requirements for publication.

The main revisions in the new manuscript are:

1. Line 42 - Edited funding resource.
2. Line 77 - Added the abbreviation of MDT.
3. Line 108 - “Lamb et al” to replace “Molly”
4. Line 134&136 - Details of the ethics approval have been reported.
5. Line 147 - Frequency of regular monitoring has been supplemented
6. Line 152 - Added the full name of “MDT”, which stands for “Multidisciplinary Team”.
7. Line 162~167 - Added detailed methods for height and weight measurement.
8. Line 206 - Added the explanations for the abbreviations of table 1.
9. Line 211 - “stature” to replace “statue”
10. Line 235 - “affecting” to replace “affect”
11. Line 252 - Added the meaning of symbol “*”
12. Line 253 - Added the explanations for the abbreviations of table 2.
13. Line 348 - The description of bone turnover markers has been revised .
14. Line 380 - the unclear sentence had been revised.
15. Line 429 - “adult” to replace “elderly”.
16. Line 432 - The limitation of measuring method was added.

Responses to reviewer 1#

1. The authors mention that height and weight were measured using standard equipment. It would be beneficial to clarify what is meant by "standard equipment." Additionally, in ambulatory patients, accurate height measurement can be challenging in DMD children due to factors such as scoliosis, increased lumbar lordosis, and contractures. Therefore, it is important to specify the percentage of patients with scoliosis, contractures, and increased lumbar lordosis. For non-ambulatory patients, it is evident that height measurement cannot be performed using standard equipment, so a detailed explanation is necessary.

Response:

Thank you for this constructive suggestion. There is some inevitable bias, but we can confirm that its impact on the accuracy of the measurements is minimal by the quality control.

In this study, a calibrated device was used to measure height and weight. Each individual was measured three times, and the average value was recorded for accuracy. Also, the children were examined for spinal health (including scoliosis and spina bifida/fracture) every six months. Because the participants in this study were all under 12 years old, most of them have healthy spine and only few of them have very mild curve that is lower than 10° (which did not meet the diagnostic criteria of scoliosis that cobb > 10°) and have little impact on measurement accuracy. Moreover, for participants with scoliosis (cobb >10°), severe contractures, or inability to stand independently, the ulnar calculation and are span have been performed for height. The exact formula and reference are as follows:

Calculation formula: height (cm) = (4.605* ulna length)+(1.308*age in years)+28.003

Reference: 1) Gauld LM, et al. Height prediction from ulna length. Dev Med Child Neurol. 2004 Jul;46(7):475-80. 2) Forman MR, et al. Arm span and ulnar length are reliable and accurate estimates of recumbent length and height in a multiethnic population of infants and children under 6 years of age. J Nutr. 2014 Sep;144(9):1480-7.

According to the reviewer’s insight advice, we have added description of our measuring methods in the manuscript, and limitation of height measurement for children with minor curvature of the spine (which is not clinically significant) had been added, we will try more comprehensive methods to reduce measurement errors.

2. The abbreviation "MDT" in line 114 should be explained.

Response：

Thank you for your kind suggestion. explanation for “MDT” has been added, which stands for “Multidisciplinary Team”.

3. Bone Mineral Density (BMD) is typically expressed in units of g/cm². The authors should clarify their use of the mg/cc unit.

Response:

Thank you for the suggestion. g/cm² is the unit for the DAX measurement, which measures flat data for standard screening, while mg/cc is the unit for the QCT measurement which provides 3D imaging data for detailed bone architecture analysis. Our BMD data were provided by QCT.

4. The authors report a vertebral fracture (VF) rate of 0% in their patients. However, asymptomatic vertebral fractures can occur in 30-50% of DMD children, especially those on glucocorticoid therapy. Therefore, annual (or more frequent if symptoms are present) monitoring with lateral spinal radiographs is recommended. The manuscript does not clearly state the frequency of routine follow-ups, nor whether asymptomatic vertebral fractures were actively investigated through lateral spinal radiographs. Without this monitoring, claiming a 0% VF rate may not be accurate.

Response:

Thank you for the suggestion. The frequency of routine monitoring had been added in our manuscript.

According to other research, the prevalent of VF in DMD patients aged 7-12 with daily oral prednisone is about 2% from UK NorthStar database. For our study, the participants were aged 2-12, which is also too young to have high VF rate, while the regular monitoring (spinal radiographs every 6 to 12 months), standarized care of our multidisciplinary team (pediatric neurology, endocrinology, radiology, cardiovascular, rehabilitation, nutrition), early intervention, and tailored therapies (including spinal management) helped children with DMD maintain muscle strength and bone health. In our center, the VF prevalent was about 2%, consistent with the prevalence in other researches.

5. Are there any patients with long bone fractures, diagnosed osteoporosis, or those receiving bisphosphonate treatment?

Response:

Thank you for the suggestion. 8 participants had a previous fracture, including ankle, elbow, femur, upper arm, tibia and nose, most of these fractures happened in their early age before GC use. 91 participants with BMD z-score < -2 were diagnosed osteoporosis. 6 participants with oral Alendronate sodium and 7 participants with zoledronic acid injection in this study.

6. In Figure 1-d, the authors should explain why height growth velocity is so low at age 3 in the DMD population.

Response:

Thank you for this question. As diagnosis of DMD is usually after 3 years old, the sample size of age 3 was relatively small which may lead to bias in results. With improvement of people’s awareness and diagnostic methods, the age of diagnosis is gradually advanced, more data before age 3 will be included in our cohort.

Responses to reviewer 2#

Line 82: What is "Molly"?

Response:

We apologize that we confused the first and last name of this researcher, thank you for finding out. We had corrected this in the manuscript.

For how many subjects was parents' stature known re. the exclusion criterion? This may be documented in Endocrine assessments but isn't typical in neurology clinic.

Response:

Just as you mentioned, there are Endocrinologists in the multidisciplinary team to conduct the growth and stature monitoring. At the time of writing, no patient at our center meets this exclusion criterion in question. Nevertheless, this was retained in the manuscript because, in endocrinology, very short stature in adult parents may be regarded as a form of short stature that deviates from the familial genetic background.

Was DXA and bone age measured at the same age in all participants? How many had multiple available?

Response:

Thank you for the questions. Patients were followed-up in our center every 6 month to one year. BMD (QCT) was not measured each visit, in accordance with guideline, we started to measure BMD around the onset of GC use, BMD was measured at least once a year. Endocrinologists will conduct bone age testing according to children's growth and development needs. In this study, all participants were measured at the same age, 291 multiple data (BMD and bone age) were available.

Results

Table 1 and 2 require a legend explaining the abbreviations

Repones:

Thank you for your suggestion, explanation for abbreviations had been added.

Figure one: why is the data point for 9 years in D so low?

Response:

Figure1-D shows the growth velocity, it indicates that children's height is still increasing but at a slower rate by the age 9. The total sample size is large, but the sample size in group age 9 might insufficient, it can lead abnormal distribution, instability and bias in the statistical results. As the number of patients in our cohort increases and more repeated-measures data are collected, a longitudinal study with a larger simple supplement and verify the results in our future study.

Line 153 - "stature" to replace "statue"

Line 170 - "affecting" to replace "affect"

Line 302: suggest "adult" as "elderly" is not typical of the DMD population.

Reponse:

Thank you for all the detailed corrections. we replace these words for more accurate statements

Discussion

Recommend combining the bone marker discussion into one rather than repeating it - the first discussion of its use in clinical care is overly strong given lack of normative pediatric data and variability; the discussion is more fairly balanced when repeated later on.

Response:

Thank you for the suggestion. This part has been condensed in the manuscript.

Comment on the limitation of not assessing contractures or scoliosis in the data which both impact height and BMI measurement.

Repones:

Thank you for your helpful advice. We have added a discussion on the impact of scoliosis on height measurement in limitation section.

The children who were followed up by our team were examined for spinal health (including scoliosis and spina bifida/fracture) every six months to one year. The participants in this cohort were under age 12, most of them did not meet the diagnostic criteria of scoliosis (cobb > 10°). A mild curvature of spine can also affect the accuracy of height measurement, although height measurement for scoliosis is difficult, we will apply more comprehensive approach to improve the accuracy of height measurement, including the use of X-ray assisted measurement.

Line 273: Unclear sentence: Earlier study rather than age? After the age of 8 years?

Response:

We apologize for the unclear expression. We had revised the statements to make the sentences clearer. The revised paragraph is as follows:

It has long been shown that in healthy individuals, peak bone mass is almost completely reached by late adolescence or early adulthood^[30]^. However, our result revealed that BMD kept accumulating during the first two years of prolonged GC exposure (aged around 5.5 to 7.5), yet after which it greatly slumped, probably influenced by both progressive myopathy and cumulative osteo-toxicity of steroids^[31]^. This was in agreement with the previous findings of our team, where we have reported that children with DMD no longer maintain their BMD leave at least age 8 years^[32]^.

PLOS authors have the option to publish the peer review history of their article ([what does this mean?](https://journals.plos.org/plosone/s/editorial-and-peer-review-process#loc-peer-review-history)). If published, this will include your full peer review and any attached files.

Response:
Yes, we agree to publish the peer review history of this article.

Thanks to the professional comments again that point out the above problems. The manuscript has revised carefully and thoroughly according to each point. We hope these explanations would answer the doubts.

Sincerely,

Dr. Xiaotang Cai
