# Supplementary material for: Height development and multiple bone health indicators in children aged 2–12 years with Duchenne muscular dystrophy (DMD)
Source: PLoS One. 2025 Jan 10;20(1):e0316938. doi: 10.1371/journal.pone.0316938 (PMC11723629; doi:10.1371/journal.pone.0316938)
Supplement: Attachment — Submitted filename: Response to Reviewers.docx [file pone.0316938.s003.docx]

**Response Letter**

Dear editors and reviewers,

Thank you for the detailed review and the precious opportunity to revise our manuscript titled “Height development and multiple bone health indicators in children aged 2-12 years with Duchenne muscular dystrophy (DMD)” (ID: PONE-D-24-27979). We are very grateful for your constructive comments and suggestions, which are very valuable and helpful for improving our manuscript. In the following, the responses to all the comments are provided one by one.

We have tried our best to make all the revisions clear, and we hope that the revised manuscript can satisfy the requirements for publication.

The main revisions in the new manuscript are:

1. Line 358 - “Tanner” to replace “Tunner”
2. Line 381 - Repeated sentence and reference had been deleted.

Responses to reviewer 2#

"Tanner' is misspelled in second use line 356.

Response:

We appreciate your careful reading of our manuscript. The misspelled word have been corrected, thank you for finding out.

The sentence starting in 376 is repeated further down the page, with a difference reference - one should be deleted.

Response:

We sincerely apologize for the oversight of repeating a sentence in the manuscript. We have removed the duplicate sentence in the revised version. Thank you for your attention to detail, which has been invaluable in improving the clarity and quality of our work.

Thank you once again to the professional comments that point out the above problems. The manuscript has revised carefully and thoroughly according to each point. We hope these explanations would answer the doubts.

Sincerely,

Dr. Xiaotang Cai
